# Food allergy and anaphylaxis – 2054. Easy-to-use severity grading system for treatment of symptoms induced by oral food challenge

**DOI:** 10.1186/1939-4551-6-S1-P137

**Published:** 2013-04-23

**Authors:** Noriyuki Yanagida, Yuu Okada, Hasegawa Yukiko, Taro Miura, Ishida Wako, Yumi Koike, Kiyotake Ogura, Katsuhito Iikura, Sakura Sato, Takatsugu Komata, Takanori Imai, Morimitsu Tomikawa, Akinori Shukuya, Motohiro Ebisawa

**Affiliations:** 1 Department of Pediatrics, National Sagamihara Hospital, Kanagawa, Japan; 2 Department of Allergy, Sagamihara National Hospital, Kanagawa, Japan; 3 Sagamihara National Hospital, Japan

## Background

The purpose of this study is to establish grading system for the evaluation of systemic reactions (SR) at oral food challengeand to investigate relationship between severity of SR using the grading system and the treatment.

## Methods

From June 2008 to June 2012, the severity of SR was assessed at double-blind placebo-controlled food challenge test (DBPCFC) to evaluate if they were candidates for rush oral immunotherapy or not. The medical records of 342 patients who showed positive reaction at DBPCFC were analyzed. A hundred and forty-one were allergic to hen's egg, 156 to milk and 45 to wheat. We modified the grading system proposed by Sampson HA in 2003 to enhance the convenience at clinical practice. It was proposed to indicate “severity of SR for each organ system, i.e., skin, mucosa, gastrointestinal tract, respiratory tract, cardiovascular, and neurological system. Systemic reactions for each organ were classified as Grade (G) 1 (mild), G2 (moderate), and G3 (severe). The severity score was based on the organ system mostly affected. We examined relationship between the severity score and its treatment during DBPCFC.

## Results

Average age of patient’s was 9.1+/-2.7y. Induced symptoms at each organ system were as follows; respiratory tract: 73%, mucosa: 68%, skin: 62%, gastrointestinal tract: 62%, neurological system: 15%, and cardiovascular system: 8%. The number of patients who showed G1, G2, and G3 was 70, 190 and 82, respectively.

Percentages of patients who used antihistamine, corticosteroid, b2 stimulant inhalation, intramuscular adrenaline were as follows; 16% (Grade1), 77% (Grade2) and 94% (Grade 3), 0% 34% 81%, 1% 62% 85% and 0% 0% 89%.

Scores over G2 was significantly increase the frequency of therapeutic intervention (> G2:93% vs. G1:17%, p<0.001, bonferroni test). G3 scores significantly related to intramuscular adrenaline administration compared to that lower than G2 sores (G3:89% vs. lower than G2:0%, p<0.001, bonferroni test).

## Conclusions

Most cases of G2 symptoms required medications and G3 symptoms did adrenaline administration. The easy-to-use grading systems according to severity of symptoms proposed here was useful to select the best approach to treat SR at OFC.

